# Covalent organic framework nanomedicines: Biocompatibility for advanced nanocarriers and cancer theranostics applications

**DOI:** 10.1016/j.bioactmat.2022.08.016

**Published:** 2022-09-14

**Authors:** Nem Singh, Jungryun Kim, Jaewon Kim, Kyungwoo Lee, Zehra Zunbul, Injun Lee, Eunji Kim, Sung-Gil Chi, Jong Seung Kim

**Affiliations:** a Department of Chemistry, Korea University, Seoul, 02841, South Korea; b Department of Life Science, Korea University, Seoul, 02841, South Korea

## Abstract

Nanomedicines for drug delivery and imaging-guided cancer therapy is a rapidly growing research area. The unique properties of nanomedicines have a massive potential in solving longstanding challenges of existing cancer drugs, such as poor localization at the tumor site, high drug doses and toxicity, recurrence, and poor immune response. However, inadequate biocompatibility restricts their potential in clinical translation. Therefore, advanced nanomaterials with high biocompatibility and enhanced therapeutic efficiency are highly desired to fast-track the clinical translation of nanomedicines. Intrinsic properties of nanoscale covalent organic frameworks (nCOFs), such as suitable size, modular pore geometry and porosity, and straightforward post-synthetic modification via simple organic transformations, make them incredibly attractive for future nanomedicines. The ability of COFs to disintegrate in a slightly acidic tumor microenvironment also gives them a competitive advantage in targeted delivery. This review summarizes recently published applications of COFs in drug delivery, photo-immuno therapy, sonodynamic therapy, photothermal therapy, chemotherapy, pyroptosis, and combination therapy. Herein we mainly focused on modifications of COFs to enhance their biocompatibility, efficacy and potential clinical translation. This review will provide the fundamental knowledge in designing biocompatible nCOFs-based nanomedicines and will help in the rapid development of cancer drug carriers and theranostics.

## Highlights

• Recent developments in cancer theranostics applications of COFs are systematically reviewed.
• The advantages of COFs over other nanomaterials in drug delivery and phototherapy are discussed.
• COF's modifications to enhance biocompatibility and efficacy in cancer theranostics are analyzed.
• Challenges and prospects for clinical translation of COFs in cancer therapeutics are debated.

## Graphical abstract

**Figure:**
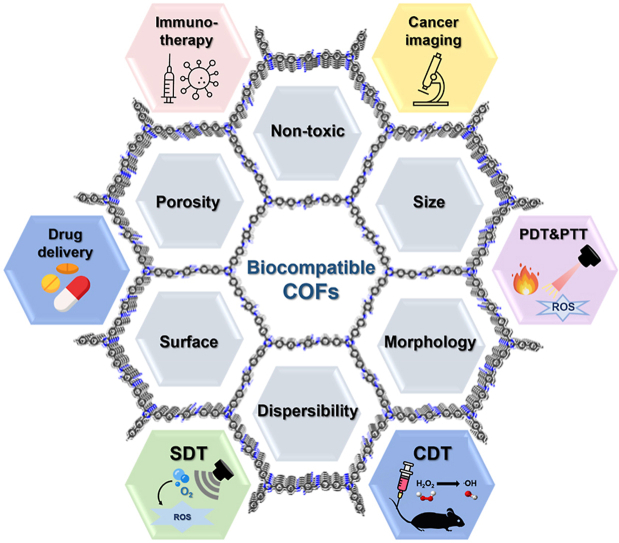

## 1 Introduction

**Fig. 1:**
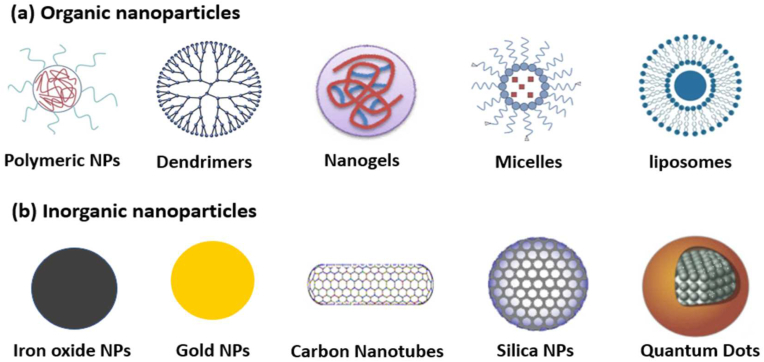
Representative examples of various traditional nanomaterials studied for cancer theranostic applications.

**Fig. 2:**
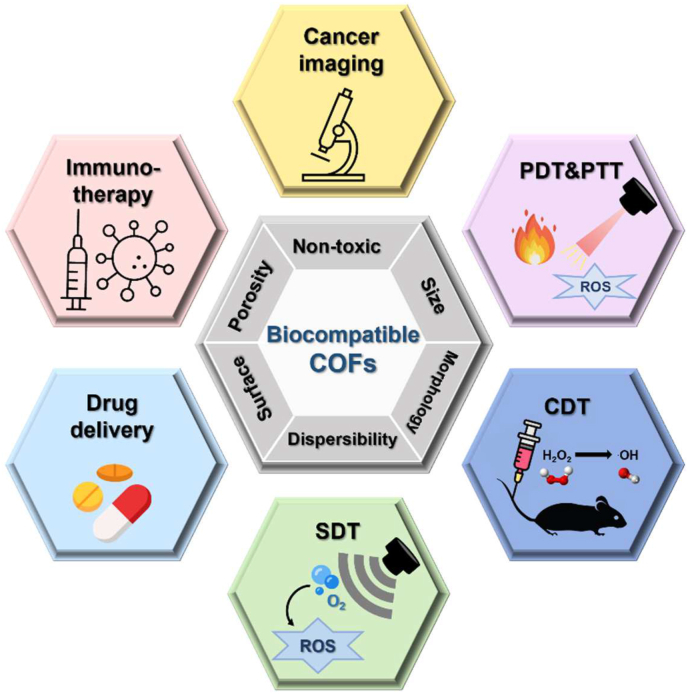
Schematic representation of biocompatible properties (i.e., ideal size, morphology, dispersibility, modular surface, porosity, and non-toxicity) and theranostic applications (i.e., drug delivery, cancer imaging, PDT, PTT, SDT, and immunotherapy) of COF NPs.

Cancer threat to human life and morbidity is continuously growing with the unavailability of effective treatments [1]. Delayed diagnosis due to inaccessible facilities is a significant cause of death in developing countries [2]. Medicinal sciences have made immense progress in finding various cancer treatments, including chemotherapy, surgery, and phototherapy. However, limitation of these treatments, such as high toxicity, long-term side effect, and recurrence due to incomplete ablation of the tumor, makes it an impenetrable challenge [3,4]. The primary cause of failing molecular chemotherapy drugs is their inability of reaching to the tumor site in sufficient quantity to induce programmed cell death. Also, due to the high toxicity of anti-cancer drugs, upon increasing the dose, it becomes highly toxic to the normal cells [5,6]. The ability of adequately sized nanoparticles to bypass the filtration process and their selective accumulation in tumor sites due to the EPR effect has presented great potential in various cancer treatments [6,7]. The early success of NPs in cancer therapy prompted intense studies to find such applications in several inorganic (silica, gold, metal-oxide, black phosphorous, quantum dots, etc.), organic, and biomolecules-based (dendrimers, mesoporous polymers, liposomes, micelles, etc.) nanomaterials (Fig. 1) [[8], [9], [10], [11]]. These nanomaterials have demonstrated promising results in various anticancer applications however, their limited functionalization ability restricts their efficiency and biocompatibility in cancer theranostic applications [[12], [13], [14]]. Moreover, the metal-based nanomaterials carry the risk of long-term toxicity; similarly, other biomaterials have the limitations of reproducibility, irrepressible morphology, and a broader range of nanoparticle size [[15], [16], [17], [18]]. Recent reports on theranostic applications of COF-based NPs in drug delivery, cancer imaging, imaging-guided therapy, photodynamic, sonodynamic and immunotherapy have demonstrated extraordinary potential (see Fig. 2).

Covalent organic frameworks (COFs) are crystalline polymers analogous to Metal-organic frameworks prepared via reticular chemistry of reversible covalent bonds [[19], [20], [21]]. The COFs are among the fastest-growing research area owing to their distinctive intrinsic properties such as modular design using abundant building blocks, simple synthetic methodology for tunable nanoparticles size for balanced stability and biodegradability, and easier post-synthetic modification for intended applications [[22], [23], [24], [25], [26], [27]]. These unique characteristics of COFs have led them to many advanced applications that include energy storage, photo and electrocatalysis, chiral separation, gas separation, dye-degradation, and biomedical applications [[28], [29], [30], [31], [32], [33], [34]]. Although the theranostic applications of COFs were started only a few years back, currently, it is growing very rapidly. As a result, some previous reviews have periodically summarized the progress in drug delivery and theranostic applications of COFs [[33], [34], [35], [36], [37], [38]]. In this review, we are explicitly evaluating the biocompatibility of COFs in theranostic applications. Moreover, we are reviewing very recent research articles opening new theranostic applications of COFs with significantly improved biocompatibility and efficacy [[38], [39], [40], [41], [42]]. We have systematically discussed recent advances of COF NPs in targeted drug delivery, photodynamic, sonodynamic, and photothermal therapy, along with immunotherapy, combination therapy, and chemodynamic therapy. We also discussed a recent example of COF NPs successfully inducing inflammatory cell death (pyroptosis) selectively in cancer cells.

## 2 Biocompatibility of COFs for anti-cancer applications

Biocompatibility is the commonly used term to describe the ability of a material to produce an appropriate host response in a specific application. Biocompatibility is also used to define the capability of the material from initiating the biological response to complete clearance without inducing unacceptable toxicity [43]. Various parameters contribute to the biocompatibility of nanoparticles like size, morphology, surface properties, pore geometry, porosity, etc. The later stage of biocompatibility is achieved when the material does not induce unacceptable toxic, carcinogenic, immunogenic responses [44]. Therefore, to assess the biocompatibility of a nanomaterial in biological applications, the physical properties of the nanoparticles must be carefully evaluated to estimate their possible interactions with blood and cell organelles. Since theranostic applications of nCOFs are in a nascent stage and the information about later stages of biocompatibility is underdeveloped, in this review, we looked at the physical properties of COFs and evaluated the biocompatibility in theranostic applications.

### 2.1 Biodegradability

The toxicity of NPs largely depends on their biodegradation mechanism, metabolization, and exclusion after accomplishing the treatment task. Recent studies have suggested the different means of degradation and clearance of nanoparticles from the cells. The exclusion rate depends on the mechanism of degradation of NPs. For example, pH-based hydrolysis of nCOFs is relatively faster than carbon nanotubes' natural enzymatic catalytic degradation [45]. To estimate the toxicity of nanomedicines, first, we need to find out how they metabolize and are excluded from the cells. Few reports have recently discussed metabolization, but the mechanism of nanomedicines exclusion is still unknown [46]. These results suggest that it is essential to consider biodegradability in terms of the rate of exclusion of nanoparticles from cells when designing nanomedicine for cancer theranostics.

### 2.2 Size

Various studies have suggested the suitable NPs size to be between 10 and 200 nm for extended blood circulation time. The NP's size should be larger than 10 nm to avoid rapid kidney filtration, whereas the size should be smaller than 200 nm to skip the liver and spleen filtration [47]. Recent research results showed that the 50–100 nm size nanoparticles exhibit improved performance *in vivo* due to greater tissue penetration and greater tumor inhibition. The conventional solvothermal synthesis usually produces the COF particles in micrometers which were not suitable for anti-cancer applications. Recently developed pre and post-synthetic modifications have resulted in the range of 50–200 nm which have been quite suitable for theranostics applications. Apart from the better cell uptake and extended circulation time in blood, NPs around 50–100 nm make up a stable colloidal solution without aggregation. Table 1 summarizes the particle size of nCOFs recently reported for anti-cancer applications.

**Table 1:** Summary of properties for biocompatible COFs.

nCOF	Morphology	Size	Strategies to regulate COFs for enhancing compatibility	Application	Reference
UC-COF	Nano-spheres	∼100 nm	Polyethyleneimine and Polyvinylpyrrolidone	PDT	[39]
LZU-1-BODIPY-2H	Nano-spheres	∼110 nm	BODIPY-2I	PDT	[119]
CaCO_3_@COF-BODIPY-2I@GAG	Nano-sheets	150 nm	Glycosamino-glycan	PDT	[120]
TAPT-DHTA-COF	Nano-dots	10 nm	PEGylation	PDT	[121]
HA@COF NSs	Nano-sheets	200 nm	Hydraulic Acid modified	PDT	[123]
COF-618-Cu	Nano-sheets	150 nm	Cu-Coordination	PDT	[124]
ICG@COF-1@PDA	Nano-sheets	130–160 nm	Polydopamine	PDT	[126]
TA–COF–P@CT	Nano-spheres	90–130 nm	PEGylation	Photosensitizer delivery/PDT	[77]
PcS@COF-1	Nano-sheets	–	PEGylation	Photosensitizer delivery/PDT	[78]
TTI–COF–Q	Nano-spheres	–	–	Anticancer drugs delivery/Chemotherapy	[85]
DOX@COF	Nano-spheres	100–150 nm	–	Anticancer drugs delivery/Chemotherapy	[86]
γ-SD/PLL	Nano-spheres	100–200 nm	poly-l-lysine modified	Anticancer drugs delivery/Chemotherapy	[56]
TPI-COF	Nano-sheets	345 nm	–	Cancer imaging	[63]
TpPa-1@Dye	Hemi-spheres	2 μm	Fluorescence dye functionalized	Cancer imaging	[65]
C–COF-survivin & C–COF-TK1)	Nano-spheres	50 nm	Carbonization	Cancer imaging	[66]
PDA@COF@DOX/IR808	Nano-spheres	185–195 nm	folic acid (FA)-F127 modified	Cancer imaging and therapy	[67]
MCOF	Nano-sphere	430 nm	COF coated on Fe_3_O_4_	Cancer imaging	[68]
COF-909-Cu	Nano-rods	150 nm	Cu-Coordination	CDT and pyroptosis	[40]
COF–TiO_2_-HA	Nano-spheres	50–100 nm	Hyaluronic Acid modified	SDT	[135]
THPP-Oxa(IV)-PEG	Nano-sheets	50–100 nm	PEGylation	SDT	[137]
CPF-Cu	2D nanocrystalline	10 nm	1,2,4,5-tetracyanobenzene modified	PTT	[144]
COF-PDA-FA	Nano-spheres	150 nm	polydopamine (PDA) and	PTT	[145]
			folic acid (FA) modified		
COF-GA	irregular morphology	100–200 nm	Gambogic acid modified	PTT	[146]
DPPN COF	Nano-spheres	200–800 nm	DPP (aldehyde monomer) and TAPA (amino monomer) modified	PTT	[149]
TPAT COF	Nano-spheres	130–600 nm	Thienoisoindigo and tris(4-	PTT	[150]
			aminophenyl) amine		
			Modified		
Fe_3_O_4_@COF(TpBD)	Micro-spheres	1.3–2.0 nm	Polyimine network coated on Fe_3_O_4_	PTT	[147]
Py-BPy^+•^-COF	2D-layer nano structure	90 nm	Py-TA and 2,2′-BPy-DCA modified	PTT	[148]
CuS@COF-BDP	Nano-particle	∼140 nm	–	PTT/PDT	[162]
Cu-DhaTph	2D-layer nano structure	∼75 nm	Cu-Coordination	PTT/PDT	[163]
CIO	Nano-spheres	∼100 nm	OVA coating	PTT/PDT/Immuno-therapy	[164]
BMCAP	Nano-squares	∼120 nm	PEGylation	PTT/PDT/Anti-Vascularization	[165]
COF@IR783@CAD	2D-layer nanoparticles	350 nm	Ultrasonic exfoliation, Drug loading	PTT/Chemotherapy	[166]
VONc@COF-Por	Nano-particle	∼140 nm	Ultrasonic exfoliation, VONc loading	PDT/PTT	[167]
RSL3@COF–Fc	Nano-spheres	180 nm	FcCHO-RSL3 modified	CDT	[158]

### 2.3 Morphology

Morphology of the nanoparticles also plays an essential role in the compatibility of nanomaterials in biological applications. Cell uptake of various morphologies such as nanospheres, nanosheets, nanotubes, nanocapsules, etc., have been applied for cancer theranostics applications. A study suggested the faster cell uptake of spherical nanoparticles than nanorods, possibly due to lower aggregation of spherical nanoparticles [48]. The surface morphology and size are estimated using a scanning electron microscope (SEM), field emission scanning electron microscope (FESEM), tunneling electron microscope (TEM), atomic force microscopy (AFM), etc. microimaging techniques. The COF NPs have been synthesized in several kinds of morphologies that include cylindrical nanotubes, nanodiscs, nanospheres, hemispheres nanosheets, nanowires, etc., Table 1 summarizes the morphology of nCOFs discussed in this review for various anti-cancer applications.

### 2.4 Surface and dispersibility

The surface properties also define the biocompatibility of NPs for biological application. For example, hydrophilic side chains on the surface increase the dispersibility in biological media. The surface charge of the NPs also plays a vital role in biocompatibility; the charged NPs are comparably more toxic to the cells than the neutral ones. Also, the neutral NPs have high blood circulation time [49,50]. Since NPs are not soluble, their dispersibility makes them applicable in biological experiments. Smaller NPs sizes (≈10–200 nm) with hydrophilic functionalities generally have sufficient dispersibility for biological applications. However, hydrophobic nCOFs need PEGylation or other modifications to add hydrophilic side chains on their surface [51]. Table 1 recapitulates the pre-or post-synthetic modifications for improving the biocompatibility of nCOFs.

### 2.5 Porosity and pore sizes

Porosity and pore geometry is crucial in drug delivery applications. The potential of drug loading can be estimated by comparing the pore geometry and size of the drug molecules. The higher surface area and the suitable pore geometry give higher drug loading efficiency. Pore geometry is easily tunable in COF nanoparticles by choosing appropriate building blocks [52,53].

### 2.6 Non-toxicity

Since most of the imine COFs are hydrolyzed to building blocks at a slightly acidic pH of the cancer microenvironment, COF nanomaterials' non-toxicity depends on the building block's minimal toxicity. Designing COF NPs using previously known non-toxic building blocks provides advantages of the relatively easy and quicker estimation of long-term toxicity of COF nanomaterial [54].

## 3 Optimization of COFs for biomedical applications

Soon after the discovery, COFs started finding applications in many fields, including separation, catalysis, photocatalysis, dye degradation, etc. [[21], [22], [23], [24], [25]]. However, their biomedical applications were not realized in the early stage. Most of the COF nanomaterial prepared via the solvothermal method were not biocompatible in terms of particle size, hydrophilicity, and dispersibility in biological media. For the last few years, the biological and anti-cancer applications of COFs have been rapidly growing owing to the advanced preparation methods and the potential of COFs for post-synthetic modifications. Prepared using just organic building blocks COFs can be easily modified using simple organic reactions to enhance efficiency and biocompatibility in anti-cancer applications. Many new protocols have been recently discovered to achieve optimum biocompatibility by pre- and post-synthetic modifications [55]. Some recent reports have shown that COFs coated on the surface of other nanomaterials such as iron oxide, porous silica, and rare earth metal nanoparticles can further enhance the efficacy in PDT, PTT, and imaging-guided therapy and targeted drug delivery [39,56]. Table 1 summarizes the recently optimized surface functionalization methods to enhance efficiency and biocompatibility. As described in Table 1 numerous efforts have been put together to enhance the biocompatibility of covalent organic framework nanomaterials and fully utilize their potential in cancer theranostic applications. Although some protocols have been developed to prepare biocompatible COFs there are still many hurdles to getting optimum biocompatibility and reaching the clinical trial stage.

## 4 COFs for cancer imaging

Bioimaging technologies have significantly quickened the pace of preliminary diagnosis and detection of various cancers [34,[57], [58], [59]]. Fluorescence imaging is a well-established technique for obtaining an accurate diagnosis and smoothing the cancer therapy process [59,60]. Due to its sensitivity, and cost-effectiveness, fluorescence imaging has become a convenient technique in cancer-related analysis. Subsequently, COFs offer an excellent bioimaging potential because of their distinguishing characteristics, like eclipsed π–π stacking structure and the long-range crystal domain [61,62]. Well-performing biosensors for efficient bioimaging have become viable due to the possibilities of pre- and post-synthetic modifications in COFs to achieve optimized fluorescence, high photostability, extended π-conjugation, and minimal toxicity.

**Fig. 3:**
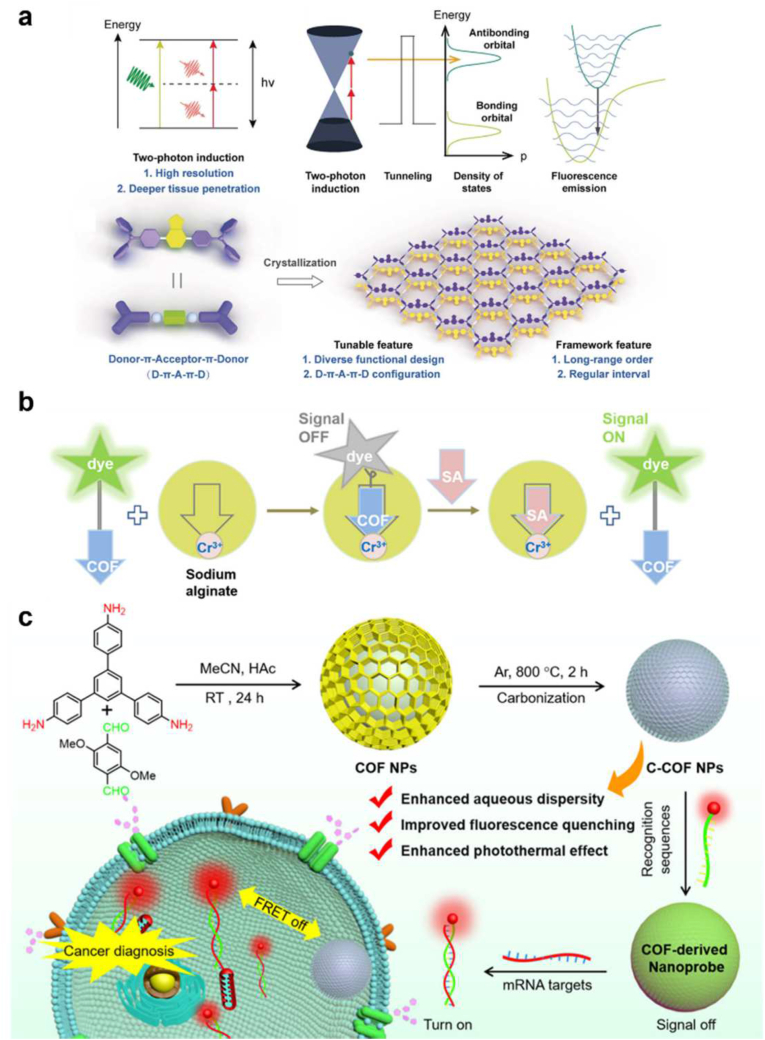
(a) Schematic illustration of one-photon induction of TPI-COF facilitating near-infrared light-induced fluorescence emission. Reproduced with permission from ref. 63. Copyright 2020, Wiley-VCH. (b) Fluorescence Turn-on Mechanism Allowing for the Detection of SA. Reproduced with permission from ref. 65. Copyright 2020, ACS publications. (c) Schematic Illustration of the Preparation of Carbonized COF-Based Nanoprobes for Cancer Cell Imaging. Reproduced with permission from ref. 66. Copyright 2021, ACS publications.

Zeng et al., for instance, demonstrated **TPI-COF**, which is COF based on benzothiadiazole for increasing two-photon induction (TPI) and obtaining two-photon promoted fluorescence emission with great efficiency (Fig. 3a) [63]. The development of COFs improved the TPI cross-section qualities significantly when the crystalline feature was obtained by having a π-conjugation domain and a framework of the matched monomer that have regular spaces of chromophore units [64]. This outperformed the results of previous conventional technologies like molecular design and polymerization. Along with the improved TPI efficiency, **TPI-COF** was employed and confirmed in malignant cellular dispersion and endocytosis process. In addition, **TPI-COF**'s biosafety and two-photon near-infrared (NIR) fluorescence imaging both *in vitro* and *in vivo* were also explored. Moreover, Wang et al. have reported using **TpPa-1@Dye** fabricated with fluorescein sodium to make hydrogels for subsequent examination of sialic acid(SA), a potential ovarian cancer biomarker (Fig. 3b) [65]. They utilized the indicator displacement assay (IDA) technique, and in IDA-in-COF system, the **TpPa-1@Dye** serves as an indicator and Cr^3+^ is an electron-deficient group that acts as a receptor. It accomplished ultrasensitive (ppb level) and a broad linear range (10^−8^−10^−2^ M) detection of SA and presented potential to cancer imaging and diagnostics because of the competitive SA and Cr^3+^ interaction. Followingly, Gao et al. fabricated COF-derived carbonous nanoprobes (**C–COF-survivin** and **C–COF-TK1**) visualizing mRNA in live cells (Fig. 3c [66]. The authors used a carbonization approach that improved fluorescence quenching effectiveness and water stability, resulting in COF derived nanoprobes that are biocompatible and multi-functional. The carbonization technique eliminated the COFs' aromatic rigid building monomers as a source of possible biotoxicity. Moreover, it could accomplish high bioimaging performance and a good photothermal conversion effect and porous structure. Gao et al. additionally reported a COF-based polydopamine core-shell nanoplatform (**PDA@COF**). Because of the porous nature of the COF, this nanoplatform demonstrated improved drug loading efficacy, numerous pores, including functional sites, and no undesired drug leakage [67]. Furthermore, the tumor targetable nanosystem was created by loading IR808 and coating F127-FA, which allowed for real-time observation using NIR fluorescence (FI), photothermal (PTI), and photoacoustic (PAI) trimodal imaging. Additionally, Liang et al. created magnetic covalent organic framework nanospheres (**MCOF**) by combining Fe_3_O_4_ nanoassemblies as cores and high-crystalline COF as shells [68]. They were able to identify miRNA-182 with sensitivity attributed to a unique interaction (fluorescence quenching or amplification) between **MCOF** and hairpin DNA. Furthermore, they used this biosensor to measure miRNA-182 from the serum of glioma patients, suggesting a reliable method for glioma detection and diagnosis/prognosis.

## 5 COFs for various cancer therapies

### 5.1 COFs for drug delivery

#### 5.1.1 Delivery vehicles of photosensitizers

The exceptional properties of nCOFs like modular porosity and pore geometry, high surface area, high porosity, and suitable morphology make them high drug-loading vehicles. Their disintegration ability at lower pH of the cancer microenvironment gives them added advantage of targeted delivery of PSs and anti-cancer drugs at the tumor site [56,[69], [70], [71]]. The intercalation of the drug molecules in the suitable pores of COF is stabilized by weak interaction forces such as hydrogen bonding, electrostatic, and van der Waals interactions with the functional groups of COFs [72]. The pore geometry of COF nanostructures to encapsulate particular drugs can be fine-tuned by choosing the building blocks of specific dimensions. Developing an efficient photosensitizer (PS) delivery vehicle can significantly improve the efficacy of photodynamic therapy (PDT) and photothermal therapy (PTT). Various functionalized COF nanocomposites have been developed to overcome the limitations of a conventional delivery system, such as reduced efficacy in hypoxia and low cell-uptake ability.

**Fig. 4:**
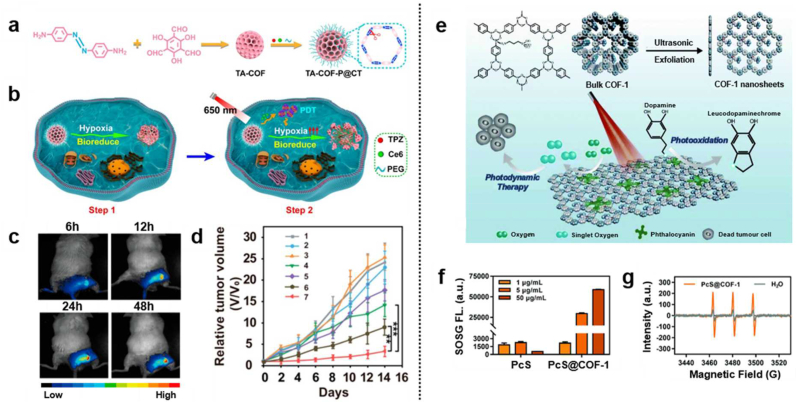
(a) Schematic illustration of the synthesis of light-induced sequential activatable TA–COF–P@CT for combined cancer therapy. **(b)** Detailed mechanism of the combined cancer therapy of TA–COF–P@CT sequential activated by light and hypoxic conditions. **(c)***In vivo* time-dependent fluorescence images of the 4T1 tumor-bearing mice after intravenous injection of TA–COF–P@CT. **(d)** Tumor growth curves during 14 days after different treatments: (5) TA–COF–P@CT (−), (7) TA–COF–P@CT (+). Reproduced with permission from ref. 77. Copyright 2021, ACS publications. **(e)** Schematic illustration of PcS@COF-1-mediated combination therapy of photooxidation and PDT. **(f)** Evaluation of ^1^O_2_ generation under different concentrations of PcS@COF-1 and free PcS. **(g)** ESR signals of ^1^O_2_ produced by PcS@COF-1 nanosheets or water upon laser irradiation as a control group. Reproduced with permission from ref. 78. Copyright 2021, The Royal Society of Chemistry.

Several strategies using nanocarriers have been suggested to overcome the limited PDT efficacy in hypoxia, thereby avoiding the limitations that conventional approaches suffer [[73], [74], [75], [76]]. In recent years, hypoxia-responsive group functionalized COF structures have been highlighted owing to their potential for medical utilities. In 2021, Jiang's group introduced light-activated and hypoxia-sensitive combined COF structure with a particle size of 90 nm for multiplicative delivery of chlorin e6 (Ce6) and tirapazamine (TPZ) with azo bond-linked as a backbone of COF (Fig. 4a) [77]. In step 1, when the hypoxia-sensitive COF structure (TA–COF–P@CT) is introduced on tumor sites, schiff base-containing COF is first degraded to release TPZ. Subsequently, under the laser irradiation in step 2, Ce6 generated reactive oxygen species(ROS) to kill the cancer cells, increasing tumor hypoxia and accelerating reductase production (Fig. 4b). Consequently, releasing rate of TPZ shows a significant increment; thereby, the cancer therapeutic effect shows outstanding improved results compared to the control group (TA–COF–P@CT (−)) (Fig. 4c and d). It would imply that TA–COF–P@CT has good biocompatibility and suitability for biomedicines and the ability to treat hypoxic tumors. In 2020, Yuan's group demonstrated COF nanosheets with high loading and therapeutic efficacy [78]. The unique design of amine-functionalized COF bulk structure with an average particle size of 130 nm has high hydrolytic stability. Subsequently, the π-π interactions with functionalized COF integrated phthalocyanine as a typical photosensitizer. They obtained the desired photosensitizer-integrated product, the PcS@COF-1 nanosheets, through the ultrasonic exfoliation (Fig. 4e). Several sequential experiments verified the evaluation of singlet oxygen (^1^O_2_) generation under 660 nm irradiation. The amount of the singlet oxygen was proportionally produced by increasing the concentration of PcS@COF-1 (Fig. 4f). The ESR study further verified the efficient singlet oxygen generation (Fig. 4g). The results demonstrated that this COF nanostructure only in a small concentration (3 μg/mL) could effectively suppress tumor growth, mediated by excellent PDT efficacy *in vitro* and *in vivo* and low cellular toxicity.

#### 5.1.2 Delivery vehicles of anti-cancer drugs

Applications of nCOFs as a drug delivery vehicle are rapidly growing, owning to their supportive intrinsic properties for efficient drug loading and selective targeted delivery at the tumor site. Notably, COFs provide the opportunity to incorporate drug molecules within the COF structure via several weak bond interactions for drug loading. Researchers have recently developed several functionalized nanocarriers for the sustainable and target-specific delivery of bio-sensitive molecules [[79], [80], [81], [82], [83], [84]]. Well-regulated and target-specific drug delivery with unique COF nanostructure has significantly improved efficacy compared to the conventional chemotherapy approaches in successful *in vivo* studies.

**Fig. 5:**
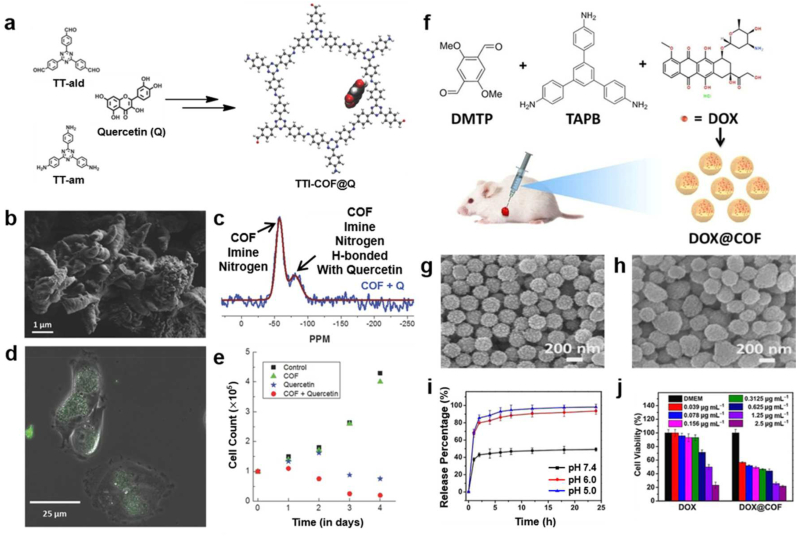
(a) Schematic illustration of synthesis of the TTI-COF from TT-ald and TT-am and Quercetin (Q)-loaded COF; TTI–COF–Q. **(b)** SEM image of the COF showing an elongated morphology **(c)**^15^N direct excitation ssNMR of the Quercentin-loaded COF (blue) spectrum fitting (brown). **(d)** The fluorescence microscope image of TTI-COF@Q uptake by MDA-MB-231 carcinoma cells **(e)** Proliferation assay of the cancer cells treated with the COF (green triangles), Quercein (blue stars), and Quercetin-loaded COF (red dots) and control group (black squares) over a period of 4 days. Reproduced with permission from ref. **85**. Copyright 2016, Wiley-VCH GmbH. **(f)** Schematic diagram of the preparation and administration of DOX@COF. SEM images of **(g)** COF, **(h)** DOX@COF. **(i)** Release profiles of DOX@COF with pH variation **(j)***in vitro* cell viability of DOX and DOX@COF aginst HeLa cells after 24 h incubaton. Reproduced with permission from ref. **86**. Copyright 2019, Wiley-VCH GmbH.

Lotsch and co-workers, for instance, presented a unique of COF (Fig. 5b) for selective uptake and targeted release of quercetin as a model drug that has anticancer and antitumor therapeutic activities of significant potency. The free electron pairs on the imine nitrogen of COF were reversibly anchoring guest molecules through non-covalent interactions [85]. Along with the non-covalent bonding, the polyphenolic nature of the molecule COF macro-structure provides an optimum platform for H-bonding interaction in the solid state and was thus expected to derive proper intercalation of quercetin into the COF pores (Fig. 5a, c). Owing to the high stability and biocompatibility of COF NPs as a nano drug carrier, a novel Quercetin-loaded COF (TTI-COF@Q) was effectively engulfed by human breast carcinoma cells and induced apoptosis. TTI-COF@Q also significantly suppressed the proliferation rate of human breast carcinoma cells compared with direct drug administration of quercetin (Fig. 5d and e).

Liu et al., for instance, suggested one-pot synthetic method of doxorubicin (DOX) @COF for the first time. DOX@COF shown enhanced antitumor efficacy through the high drug-loading capacity and pH-responsive drug release property. Simple condensation between 1,3,5-tris(4-aminophenyl)benzene (TAPB) and 2,5-dimethoxyterephthaldehyde (DMTP) as an original procedure of preparation of COF structures was slightly changed. They suggested that one-pot synthetic route of DOX@COF through DOX and DMTP were thoroughly mixed, then TAPB was added to construct the Schiff base, which finally formed DOX@COF (Fig. 5f). Although the crystallinity was slightly decreased due to the reaction and Schiff base formation between DOX and DMTP (Fig. 5g), homogeneous morphologic construction (Fig. 5h) and excellent drug loading efficiency have proven to fit to be utilized for anti-cancer drug delivery [86]. Indeed, high biocompatibility carry-over from pH-responsivity of Schiff base (Fig. 5i), could appropriately induce cancer therapeutic effect by DOX@COF, and prevent the overdose compared with free DOX administration (Fig. 5j).

**Fig. 6:**
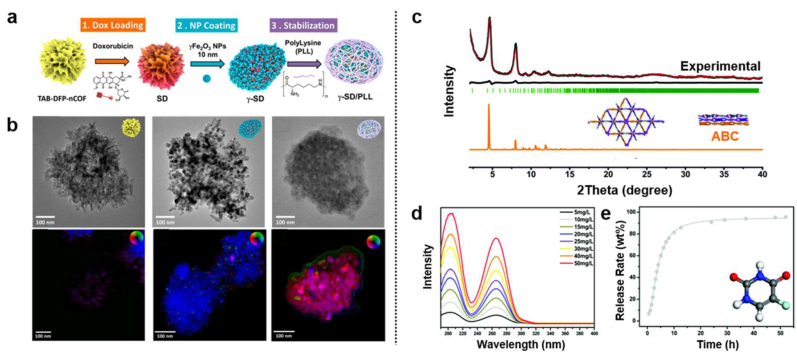
(a) Schematic representation of γ-SD/PLL synthesis. (b) HRTEM and Differential phase contrast (DPC) STEM images of SD, γ-SD and γ-SD/PLL. Reproduced with permission from ref. 56. Copyright 2020, ACS publications. (c) PXRD graph of Cage–COF–TT; experimental (black), simulated ABC stacked (orange) (d) UV–vis spectra of 5-Fluorouracil (5-FU) in simulated body fluid (SBF; pH 7.4 buffer solution) at different concentrations. (e) Time-dependent drug releasing profile of 5-FU-loaded Cage–COF–TT. Reproduced with permission from ref. 90. Copyright 2021, The Royal Society of Chemistry and Cetre National de la Recherche Scientifique.

In 2020, Trabolsi's group constructed a multi-functional magnetic COF, TAB-DFP-nCOF and successfully applied as MRI, chemotherapy and hyperthermia agents [56]. First, they synthesized this COF under microwave irradiation at 110 °C for 30 min using 1,3,5-tris(4-aminophenyl)benzene and 2,6-diformylpyridine (DFP) building blocks with an average particle size of ∼240 nm. Subsequently, the anti-cancer drug DOX was loaded on the TAB-DFP-nCOF, further iron oxide nanoparticles were loaded to it, and finally, poly-l-lysine (PLL) was coated on the surface, which has the ability of selective internalization into cancer cells. The PLL coating not only stabilized the magnetic nanoparticles but also improved dispersibility and compatibility in the complex biological system (Fig. 6a and b). Due to the acidic pH-sensitive imine bond, the COF particles disintegrate, losing their typical shape under the acidic media such as lysosomal degradation through the natural pathway of endocytosis, eventually releasing drug [[87], [88], [89]]. Furthermore, intrinsic characteristics of γ-Fe_2_O_3_ NPs, magnetism can accelerate cancer therapeutic effect and magnetic resonance imaging and hyperthermia therapy with alternating magnetic field (AMF). As a result, multimodal magnetic nCOF significantly reduces cancer cell selectively over the noncancerous cell, HEK293. In 2021, Feng's group reported a Cage–COF–TT structure as a drug caging system [90]. Prism-like organic molecular cage with a Two-dimensional (2D) porous Cage-COF based on a diamond network with hexagonal vertices has a pore size of 10 Å, showing excellent drug loading performance. The structure of Cage–COF–TT was verified as exclusive ABC stacked models with PXRD patterns (Fig. 6c). They successfully applied this COF in the loading and controlled release of anti-cancer drugs 5-fluorouracil. This COF nanostructure was confirmed as an efficient drug delivery system with good biocompatibility (Fig. 6d). Furthermore, Cage–COF–TT showed no significant cytotoxicity in the 0–500 μg mL^−1^ concentration, showing COF's potential as nanomedicine. To improve the biocompatibility and therapeutic effect nCOFs on tumors, biochemical properties were optimized such as cellular permeability, hydrolytic stability, and bio-responsivity through modification of their chemical structures and surface coating giving good tumor targeting ability.

Metal-organic frameworks (MOFs) and COFs, both are crystalline porous materials with tunable pore volumes and surface properties, the unique intrinsic properties MOFs have demonstrated tremendous potential in several advanced applications. Being metal as a major constituent of their networks, MOFs for anti-cancer applications are not as favorable as other applications due to the possibility of metal-induced toxicity. Metal-free crystalline porous network of COFs offers higher biocompatibility for drug delivery of photosensitizers and anti-cancer drugs.

### 5.2 COFs for PDT-driven cancer immunotherapy

Most modern cancer treatment procedures, surgery, radiation therapy, and chemotherapy rely on eliminating cancer cells, which also causes organ malfunction and cellular imbalance. In addition, chemotherapies also face damaged healthy cells and have problems with drug resistance and cancer recurrence [[91], [92], [93]]. Cancer Immunotherapy, which enhances the body's immune system to kill cancer cells, has made considerable advancements in oncology [[94], [95], [96]]. Recent advances in cancer treatment have centered on modulating the immune response against cancer cells. This advancement has been driven mainly by cancer cells evading immune regulation, widely applied to tumors resistant to traditional therapy. The appropriate immune system is critical in cancer prevention, progression, and treatment. Although the function of the immune system in cancer therapy has been deeply studied, current anti-cancer therapy has embraced the premise that contact between dying or dead cancer cells and immune cells is a critical aspect in determining the efficacy of cancer treatment [[97], [98], [99]]. Throughout the tumor growth, many point mutations accumulate and structural changes occur in genome, resulting in genomic instability and cancer. Tumor antigens are possibly produced due to such genetic changes, and the immune system could detect them as foreign substances and mount an immunological response. Adaptive and innate immune system cells enter the tumor microenvironment (TME) and modulate tumor growth, making the immune system critical to immunosurveillance. Effective immune responses might eliminate cancer cells or damage their morphologies and activities. However, cancer cells have developed numerous mechanisms to avoid immune surveillance, including defects in antigen presentation patterns, resulting in impaired immune cell proper function and suppressed anti-cancer immune responses [100]. The interaction between cancer cells and their microenvironment, particularly its immunological components, is critical to the formation and progression of human neoplasms. Immunosurveillance is typically carried out by type 1 CD4^+^ T-helper (TH1) cells and CD8^+^ cytotoxic T lymphocytes (CTLs), which identify antigenic epitopes that emerge during malignant transformation and tumor growth [97].

The development of cancer immunotherapy was motivated mainly by cancer cell escape from immunological regulation and, as a result, tumor resistance to traditional treatments. One of the most promising concepts to eliminating tumor cells is immunogenic cell death (ICD) [94]. Particularly, ICD is followed by the exposure and release of multiple damage-associated molecular patterns (DAMPs), which give a substantial adjuvanticity to dying cancer cells by promoting the recruitment and activation of antigen-presenting cells. Many bioactive molecules are released by stressed and dying mammalian cells. In their normal state, these molecules are kept inside cells and play an important role in their proper operation. However, when they are released into the environment, they operate as alarm signals and can be detected by both the innate and adaptive immune systems. Calreticulin (CRT), heat shock proteins (HSPs) 70 and 90, high-mobility group box 1 (HMGB1), secreted ATP, annexin A1 (ANXA1), type I interferons (IFNs), and mitochondrial DNA are all on the list of DAMPs, which is still expanding. Because ICD generates anti-cancer immune responses that are necessary for the effectiveness of cancer treatment as well as for long-term anti-cancer immunity, the potential of cancer therapy to trigger ICD is therapeutically significant [101,102]. Many researchers are now focused on ICD, which may be triggered by a variety of triggers and anti-cancer treatment methods, including intracellular pathogens, conventional chemotherapy, targeted anti-cancer agents, radiotherapy, various forms of irradiation, oncolytic viruses, and PDT. The DAMP profiles of ICDs caused by different stimuli can be different, and ICDs have also been linked to different types of cell death, such as apoptosis, necroptosis, and ferroptosis [94,97,[103], [104], [105]].

Phototherapy, in addition to chemotherapy, is a prominent therapeutic strategy for the treatment of many malignancies. Using a photosensitizer, a non-toxic and light-sensitive dye, PDT kills cancers by generating ROS. PDT is considered a highly safe and spatiotemporally controlled therapy because to the great biocompatibility of PSs and the exciting laser's outstanding controllability [[106], [107], [108]]. After the PS accumulates preferentially in the tumor, it is activated by a suitable wavelength of visible light [109]. PDT has been shown in many studies to be a potent modulator of both innate and adaptive immunity [[110], [111], [112]]. PDT-induced local damage and oxidative stress in tumor sites initiate an initial inflammatory response required to eliminate tissue residues and restore homeostasis. However, PDT-induced ICD activates antitumor immunity through danger signaling systems including DAMPs, which stimulate innate immunity and activate adaptive immune responses [96]. Thus, ICD refers to the activation of innate and adaptive immune system components by DAMPs produced actively or passively. The most common feature of dying PDT-treated cancer cells, for example, is the exposure of the calcium-binding protein CRT on the plasma membrane's outer surface. CRT is generally found in the ER lumen, but when exposed on the surface, it is detected by LPR1, CD91, and acts as a ‘eat me’ signal for antigen-presenting cells (APCs) [104,[113], [114], [115]].

**Fig. 7:**
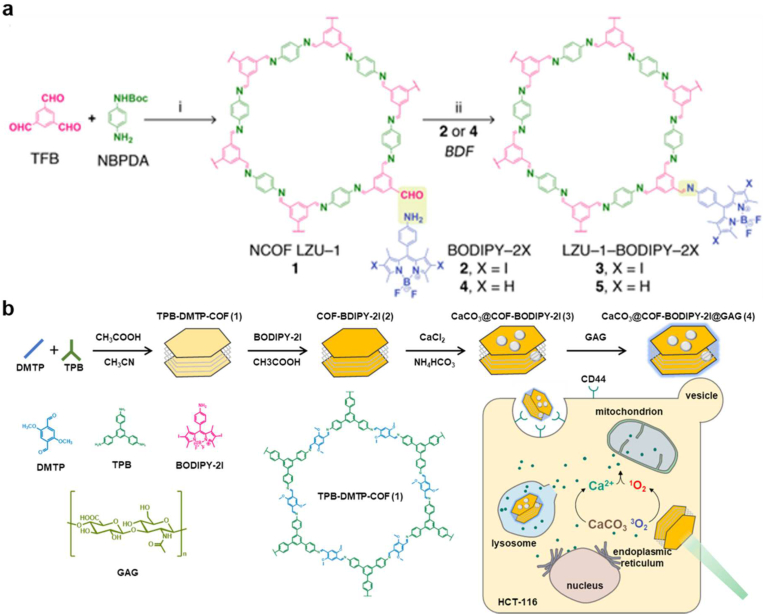
BODIPY-based COFs. (a) Synthesis of LZU-1-BODIPY. Reproduced with permission from ref. 119. Copyright 2019, Elsevier. (b) Synthesis of CaCO_3_@COF-BODIPY-2I@GAG.

Drawbacks of traditional PSs (such as porphyrin and BODIPY) such as aggregation in physiological conditions, inadequate accumulation at the tumor site and poor immune response restrict the potential of PDT in complete eradication of cancer [116,117]. Recent literature has shown that nCOFs are potential photosensitizers capable of inducing a long-term immune response. In 2019, Guan et al. for first time reported a BODIPY-modified COFs having excellent anti-cancer PDT efficacy *in vitro* and *in vivo*. The COF named NCOF LZU-1 was prepared by solvothermal method using benzene-1,3,5-tricarbaldehyde and *tert*-butyl (4-aminophenyl)carbamate as monomers via imine condensation. NCOF LZU-1 was further modified by covalently attaching two amino-decorated BODIPY molecules, BODIPY-2I and BODIPY-2H with the free end –CHO groups (Fig. 7a). The resulting LZU-1-BODIPY-2I showed good biocompatibility and significantly inhibited HeLa and MCF-7 cell viability under green LED illumination compared to unmodified NCOF LZU-1, although the BODIPY concentration was very low. Remarkably, LZU-1 showed the weakest inhibition toward MCF-10A normal cells compared with other common nanomaterials, indicating that COFs might feature better biocompatibility and suitability for biomedical applications [112,118,119]. In 2020, the same group developed a COF-based nano agent, namely CaCO_3_@COF-BODIPY-2I@GAG, for synergistic cancer therapy, by equipping 1,3,5-tris(4-aminophenyl)benzene(TAPB)-2,5-DMTP-COF with a heavy atom substituted BODIPY-2I photosensitizer, CaCO_3_ NPs, and glycosaminoglycan GAG targeting agents via stepwise modification (Fig. 7b). CaCO_3_@COF-BODIPY-2I@GAG consists of CaCO_3_ nanoparticle (NP) surface-coated with BODIPY-2I as a PS and glycosaminoglycan (GAG) targeting agent for CD44 receptors on digestive tract tumor cells. The light-activated ^1^O_2_ not only kills the tumor cells directly but also causes mitochondrial malfunction and Ca^2+^ excess in them. PDT and Ca^2+^ overload synergistic therapy both improves antitumor efficiency [120].

**Fig. 8:**
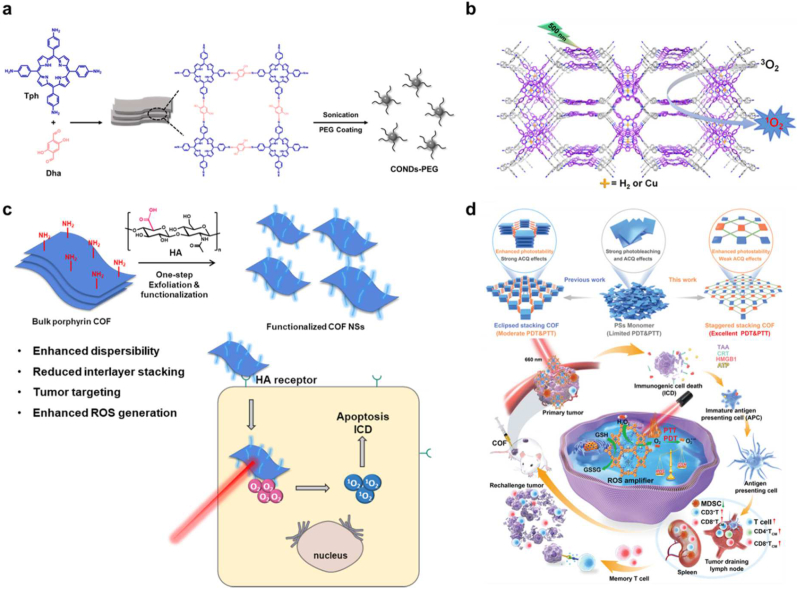
Porphyrin-based COFs. (a) TAPT-DHTA-COF. (b) 3D porphyrin-based COFs. Reprinted with permission from ref. 121. Copyright 2017 ACS publications. (c) Carboxyl-rich hyaluronic acid (HA) on porphyrin COF nanoparticles (HA@COF NSs). (d) Schematic illustration of COF-618-Cu for antitumor effect. Reproduced with permission from ref. 124. Copyright 2022 Wiley-VCH GmbH.

**Fig. 9:**
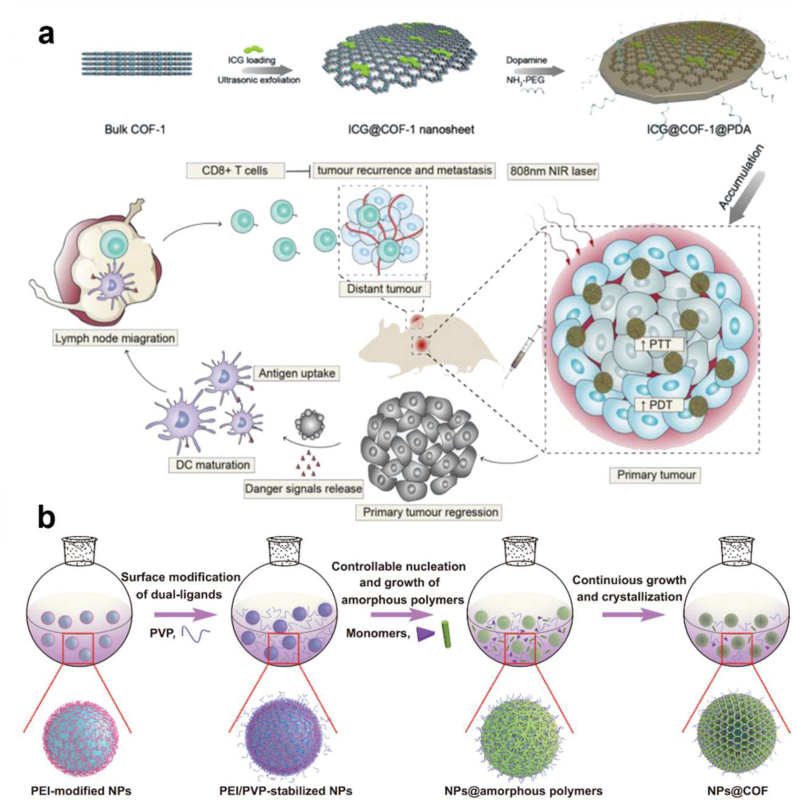
(a) ICG@COF-1@PDA. A 2D COF nanosheet with loaded photosensitizer ICG. Reprinted with permission from ref. 126. Copyright 2017 Wiley. (b) Schematic representation of mono dispersion of COF-coated NPs. Reproduced with permission from ref. 39. Copyright 2021 Nature Research.

Furthermore, Zhang et al. synthesized ultrasmall porphyrin-based COF nanodots (TAPT-DHTA-COF) and utilized them as highly effective PDT agents for cancer therapy (Fig. 8a). Well-isolated porphyrin molecules on the framework endowed the COF nanodots with a good light-triggered reactive oxygen species production ability under 638 nm irradiation, resulting in improved PDT efficiency *in vitro* and *in vivo*. In particular, the COF nanodots may be removed from the body by renal filtration without producing long-term toxicity because of their ultrasmall size (3–4 nm) [121]. Lin et al. reported the targeted synthesis of two 3D porphyrin-based COFs (3D-Por-COF and 3D-CuPor-COF), starting from tetrahedral (3D-Td) and square (2D-C4) building blocks connected through [4 + 4] imine condensation reactions (Fig. 8b). Under photoirradiation, both 3D COFs are photosensitive and may function as heterogeneous catalysts for singlet oxygen production. Compared to 3D-CuPor-COF, 3D-Por-COF has higher photocatalytic activity, showing that the characteristics of 3D porphyrin-based COFs can be adjusted by metalation of porphyrin rings [122]. Recently, Gao et al. developed ultrathin 2D functionalized covalent organic framework nanosheets (COF NSs). The author emphasized Ultrathin and even single-layered nanosheets (NSs) of porphyrin COF however, it didn't increase ROS generation, probably due to poor biocompatibility. Authors modified the NSs with carboxyl-rich hyaluronic acid to get HA@COF NSs nanoparticles which concurrently enhanced the water dispersibility and tumor cell selectivity of these NPs *in vitro* and *in vivo* (Fig. 8c) [123]. Traditional PSs such as porphyrin, chlorin e6, and indocyanine green in an aqueous solution combine to quench their fluorescence and disintegrate during laser irradiation (photobleaching), resulting in low levels of ROS generation. Consequently, it doesn't remain easy to concurrently mitigate photobleaching and aggregation-caused quench (ACQ) effects to achieve the desired phototherapy efficacy. Moreover, the distinct TME, defined by low oxygen condensation (hypoxia), low pH values, and overexpressed glutathion (GSH), is advantageous for tumor growth, invasion, and metastasis, but inhibits ROS production. Zhang et al. developed a new porphyrin-based staggered stacking COF, COF-618-Cu, that successfully reduces photobleaching and ACQ effects (Fig. 8d). COF-618-Cu can also utilize the endogenous hydrogen peroxide to generate enough oxygen to treat tumor hypoxia [124]. NIR dyes, such as indocyanine green (ICG), have promising advantages for PDT and PTT because of their remarkable optical characteristics [118,[124], [125], [126]]. There are several hurdles to overcome before PDT using NIR dyes can treat cancer. These include hypoxic tumor microenvironments and the self-quenching of photosensitizers. Nanocarriers are commonly used to transport NIR dyes because of their extremely short half-lives and poor tumor accumulation. To avoid intermolecular stacking interactions, ICG may be spontaneously adsorbed to COFs through π–π conjugations. Gan et al. described a 2D COF nanosheet with loaded photosensitizer ICG, designated ICG@COF-1@PDA, which was generated by loading ICG in COF-1 nanosheet through ultrasonic exfoliation and then coating it with polydopamine (PDA) (Fig. 9a) [126]. ICG@COF-1@PDA, when exposed to 808 nm NIR laser irradiation, generated ROS, induced inflammatory cell death, and triggered antitumor immunity in colorectal cancer. Additionally, it suppressed untreated distant tumors and metastasis of 4T1 cancers ranging from breast to lung.

Several post-synthetic modification strategies using hydrophilic chain molecules have been used for sustainable biocompatibility and targeting specific cellular organelles. These approaches have been quite successful in achieving enhanced efficiencies. Chen et al. recently reported a pre-synthetic approach to getting biocompatible COF NPs and successfully applied it to in-vivo PDT application (Fig. 9b) [39]. They in-situ coated COF layers of variable thickness over SiO_2_, metal oxide, and upconversion nanoparticles premodified with hydrophilic chains of polyethyleneimine and polyvinylpyrrolidone to increase the dispersibility in biological media. As a proof of concept, the authors in-situ layered the porphyrin-based COF over the upconversion nanoparticles resulting in a NIR activatable nano platform, UC-COF, for PDT. They successfully conducted the *in-vivo* reduction of tumors in the mice model by intravenously injecting UC-COF and 980 nm laser irradiation.

### 5.3 COFs for pyroptosis

Pyroptosis is an immunogenic programmed cell death that has recently been shown to be an effective cancer-fighting technique due to its capacity to stimulate anti-cancer immune responses by generating abundant DAMPs. The connection between pyroptosis and cancer is intricate, and the ways in which pyroptosis affects cancer differ based on the tissues and genetic code of the cancerous cells. On the one hand, pyroptosis can prevent the formation and incidence of tumors; on the other, as a kind of proinflammatory death, pyroptosis can foster the development of tumors by creating an environment that is favorable for the proliferation of tumor cells [[127], [128], [129]]. Therefore, pyroptosis can effectively promote programmed cancer cell death and is a practical anti-cancer approach *in vitro* and *in vivo* [130].

**Fig. 10:**
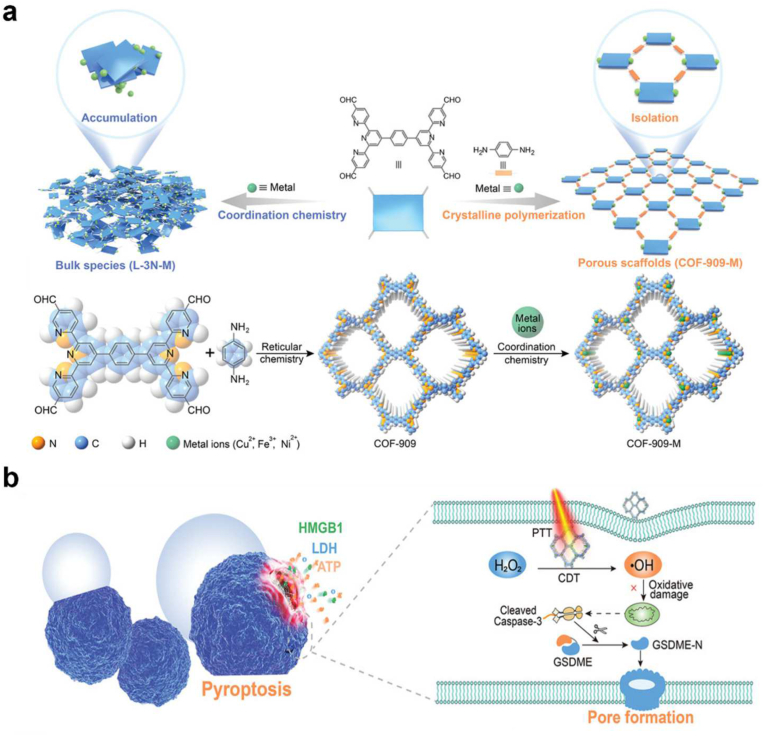
(a) Illustration of construction of multienzyme-mimicking metal modified COF-909 **(b)** Schematic illustration of the pyroptosis-inducing mechanism elicited by enzyme-mimicking COFs. Reproduced with permission from ref. 40. Copyright 2022, Wiley-VCH.

Tang et al. presented COFs that mimic several enzymes as H_2_O_2_ homeostasis disruptors that could successfully boost intracellular H_2_O_2_ levels as the first demonstration of a pyroptosis inducer based on COF (Fig. 10b) [40]. As a result, excellent chemodynamic therapy (CDT) performance and strong pyroptosis, with good pyroptosis-inducing capability, were achieved for effective cancer immunotherapy. In addition, they showed that incorporating metal ions into COF-909 scaffolds provides a novel way for fine-tuning their optical properties, such as light absorption, band energy, and stability. Metal modified COF, COF-909-Cu demonstrated reasonable biocompatibility to effectively generates pyroptosis with excellent CDT efficacy (Fig. 10a).

### 5.4 COFs for SDT

The nCOFs continuously find new applications to expand their universe in cancer therapies; sonodynamic therapy (SDT) is the latest additive to its growth potential. PDT is among the most successful and widely used techniques in cancer eradication; however, the penetration depth limitation hampers its full potential by reducing the effective half-life and radius of ROS generated during treatment [131]. SDT, with enormous preclinical and clinical potential, offers exceptional benefits in eradicating deep-rooted tumor tissue due to the ultrasound's deeper penetrability, even at a low frequency. Reactive oxygen species such as singlet oxygen and hydroxyl radical can be generated by ultrasonic irradiation, causing cancer cell death with minimal adverse effects [132]. However, developing high efficacy sonosensitizers with adequate stability remains challenging.

Organic sonosensitizers such as protoporphyrin (PpIX) and tetracarboxyphenylporphine (TCPP) were commonly utilized in the SDT process [133,134]. Nevertheless, Organic sonosensitizers are often unstable chemically and have a limited blood circulation time. Titanium-based inorganic materials have proven better sonosensitizers due to their chemical stability and low phototoxicity than organic compounds under SDT.

**Fig. 11:**
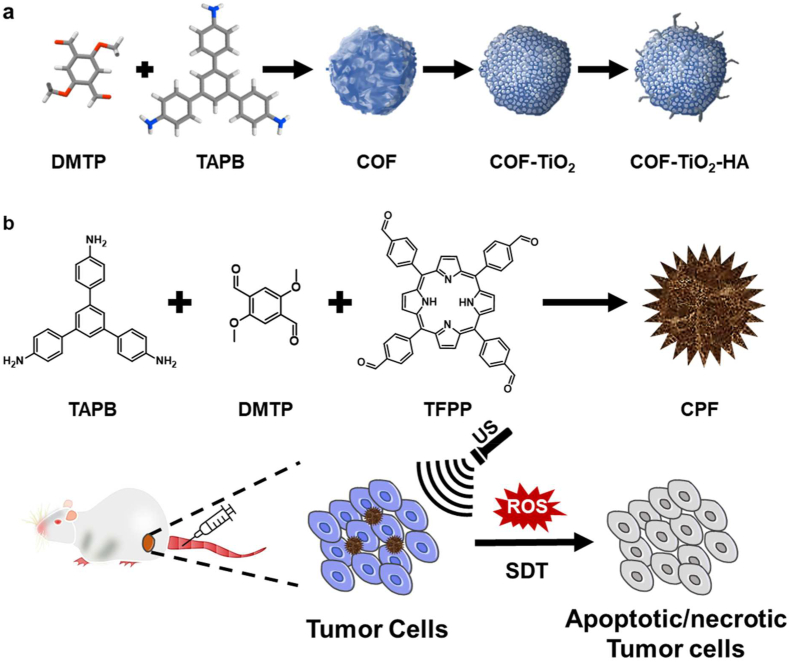
(a) Characterization of COF–TiO_2_ and COF–TiO_2_-HA. **(b)** Illustration of the synthesis and application of CPF.

Liu et al. studied the TiO_2_ to provide covalent organic frameworks, **COF–TiO**_**2**_ [135]. They have synthesized highly monodispersed COF-NPs by aldehyde amine condensation between TAPB and DMTP. **COF–TiO**_**2**_ nanocomposite was then formed by growing TiO_2_ NPs on the surface of COF *in situ*. The resulting **COF–TiO**_**2**_ was further modified with HA to get **COF–TiO**_**2**_**-HA** and achiev**e** biocompatibility (Fig. 11a). Usually, pure TiO_2_ has a wide band gap which limits the SDT effect; however, it is also known that doping with metals such as Au and Fe could reduce the band gap of TiO_2_. In this study, researchers first used **COF–TiO**_**2**_ as a sonosensitizer for lowering the band gap of TiO_2_. Upon US irradiation **COF–TiO**_**2**_**-HA** induced significantly enhanced sonodynamic impact compared to pure TiO_2_ in both *in vitro* cell viability assays and *in vivo* experiments with exceptional levels of ROS generation. The TEM and HRTEM analysis confirmed that the average size of **COF** and **COF–TiO**_**2**_ are 200 nm and 300 nm, respectively. Before exposing the US, cell viability through MTT assay was confirmed in two different cell lines. In addition, *in vivo* test shows that in the group of mice in which **COF**, **COF–TiO**_**2**_, **COF–TiO**_**2**_**-HA** were injected without using ultrasound, there was no difference in body weight, so it was judged that there was no issue with biosafety. **COF–TiO**_**2**_**-HA** seems to have good biocompatibility; however, the authors did not conduct a long-term toxicity experiment.

On the other hand, the same researchers also provided another COF-mediated sonosensitizer using porphyrin [136]. As the most often utilized organic small molecule sonosensitizers, porphyrin and its derivatives have shown high ultrasound sensitivity and are valuable in sonodynamic treatment. However, the future use of these small organic compounds is limited due to their poor biocompatibility and pharmacokinetics, low stability, and quick elimination *in vivo*. To overcome the aforementioned issues and produce the optimal therapeutic results, nanocarriers are generally used to encapsulate these small organic molecules. As a result, for the first time, the researchers created a porphyrin-incorporated COF sonosensitizer as an alternative to nanocarrier. TAPB-DMTP-COF (**CPF**) was obtained by dissolving TAPB, DMTP, 5,10,15,20-tetra(4-formylphenyl)porphyrin (TFPP) and acetic acid in a mixed solvent of acetonitrile (Fig. 11b). Under ultrasonic irradiation, the as-prepared nCOFs were capable of producing singlet oxygen. They have investigated the cell viability assay at the cellular level, and the curve of CPA nanoparticles without ultrasound shows no significant toxicity; as a result, it can be considered biocompatible.

**Fig. 12:**
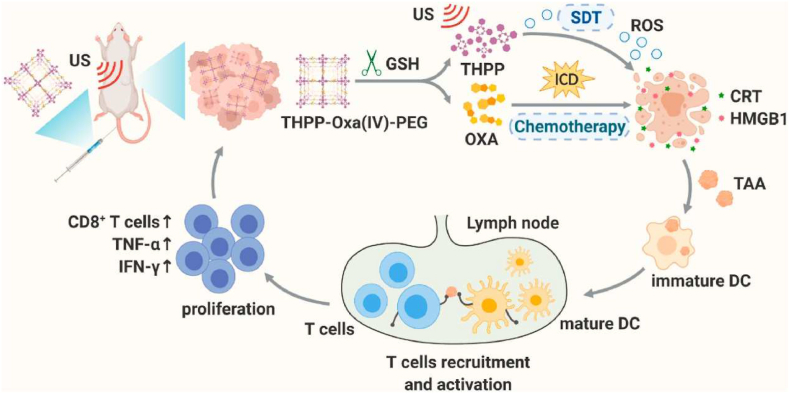
A scheme illustrating the antitumor mechanism of THPP-Oxa(IV)-PEG. Reproduced with permission from ref 137. Copyright 2022, Elsevier.

In addition to SDT using COF, Shen et al. developed a study that applied immunogenic Cancer Therapy together using GSH responsive prodrug, oxaliplatin. GSH-responsive nanomedicine was synthesized by esterifying a sonosensitizer with GSH-responsive Oxa(IV)SA_2_ in the presence of PEG_5k_-COOH. As a result, the nanoscale **THPP-Oxa(IV)-PEG** with high water stability, GSH responsive oxaliplatin release, and effective sonosensitization efficiency were produced. It was also discovered that when **THPP-Oxa(IV)-PEG** was internalized by murine CT26 CRC cells, it promoted intracellular ROS generation, which led to efficient immunogenic cell death of these cells when exposed to low-frequency ultrasound (Fig. 12) [137]. Usually, the therapeutic efficacy of sonodynamic therapy is severely limited by hypoxia in the tumor microenvironment, which is aggravated by elevated (GSH) levels in cancer cells [138]. However, the researchers offer a comprehensive strategy for developing theranostic COFs-based nanomedicine with a combination of sonodynamic and chemotherapies.

### 5.5 COFs for PTT

PTT is a form of phototherapy in which tumor cells are selectively killed by elevation in temperature upon a lower wavelength of visible light irradiation on the photothermal therapy agents (PTAs). Several PTAs like metal nanostructures, carbon nanomaterials, and conjugated polymers can employ PTT [139]. Compared to other phototherapies, several competitive advantages of PTT, such as microscopic invasion, minimal pain, and negligible slide effects, make it an efficient therapy for some selective cancers [140,141]. In addition, unlike PDT, PTT does not require oxygen, which is particularly useful in treating hypoxic tumors [142,143]. Recently, some COFs have been discovered that can generate heat upon eradiation and can also be used as PTAs in hp. Although COFs were once thought to be challenging to use in biological applications due to their bulky size and poor dispersibility in biological media. Some newly developed advanced synthetic approaches to prepare hydrophilic COFs with smaller particle sizes enhanced their compatibility for bioapplications. Li et al. synthesized a new COF, CPF-Cu, by replacing 1,2,4,5-tetracyanobenzene with 2,3-dicyanohydroquinone (DCH) with good dispersibility and biocompatibility for anti-cancer applications. The researchers revealed that CPF-Cu inhibited cancer cell proliferation and induced apoptosis in photothermic cancer therapy [144]. In addition, Song et al. introduced that delivery of glucose oxidase (GOx) using COFs with donor-acceptor structures can increase the selectivity of targeted cancer cells, and these COFs were modified with PDA and folic acid (FA) to enhance the biocompatibility [145].

**Fig. 13:**
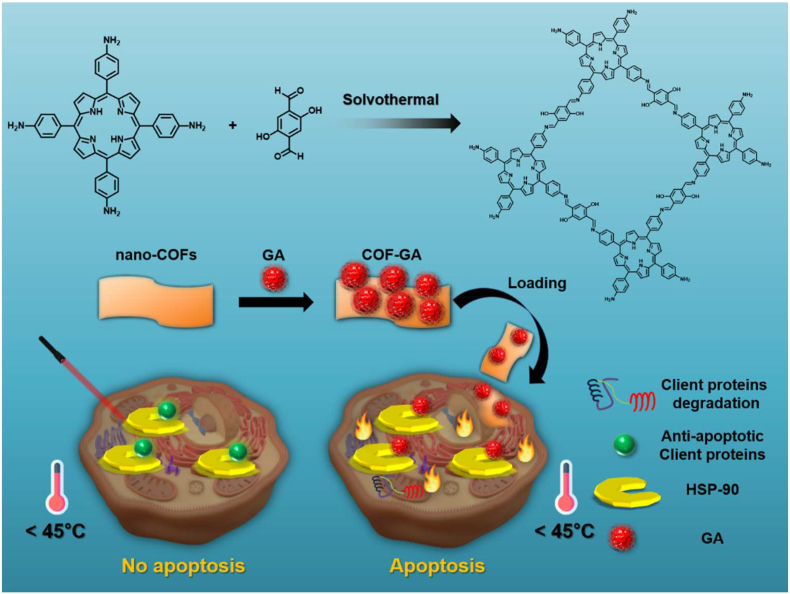
Scheme of preparing COF-GA and enhancing low-temperature PTT.

Sun et al. recently synthesized porphyrin-based COF, further modified with an HSP90 inhibitor, gambogic acid (GA) [146]. The nanomaterial COF-GA showed a moderate photothermal effect upon lesser irradiation (0.3 W cm^−2^, 635 nm, 10 min). The *in vitro* and *in vivo* results showed that even the mild PTT effect of the modified COF-GA could successfully kill the cancer cells and cure the tumor by laser irradiation (0.3 W cm^−2^, 635 nm, 10 min). Moreover, Gambogic acid can selectively prevent the thermoresistance of cancer cells by inhibiting HSP90 by directly sticking to HSP90 (Fig. 13). Also, the successful PTT efficacy in *in vivo* tests has proved the biocompatibility of COF-GA and its suitability in biomedical applications.

Similarly, Wang et al. constructed a core-shell microsphere consisting of an Fe_3_O_4_ nanocluster core and an amorphous polyimine network shell using template-mediated precipitation polymerization [147]. Under solvothermal control, they rearranged the polyimine network into imine-linked COF as shells by polymerization of monomers benzidine (BD) and 1,3,5-triformylphloroglucinol (Tp), named Fe_3_O_4_@COF(TpBD), revealing excellent photothermal conversion. By enhancing the π-electron conjugation within the 2D layers, it allows the fast transformation of NIR energy to local heat. The PEG-modified COF hybrid microspheres are found to be sustainable in the physiological conditions, ensuring outstanding biocompatibility.

**Fig. 14:**
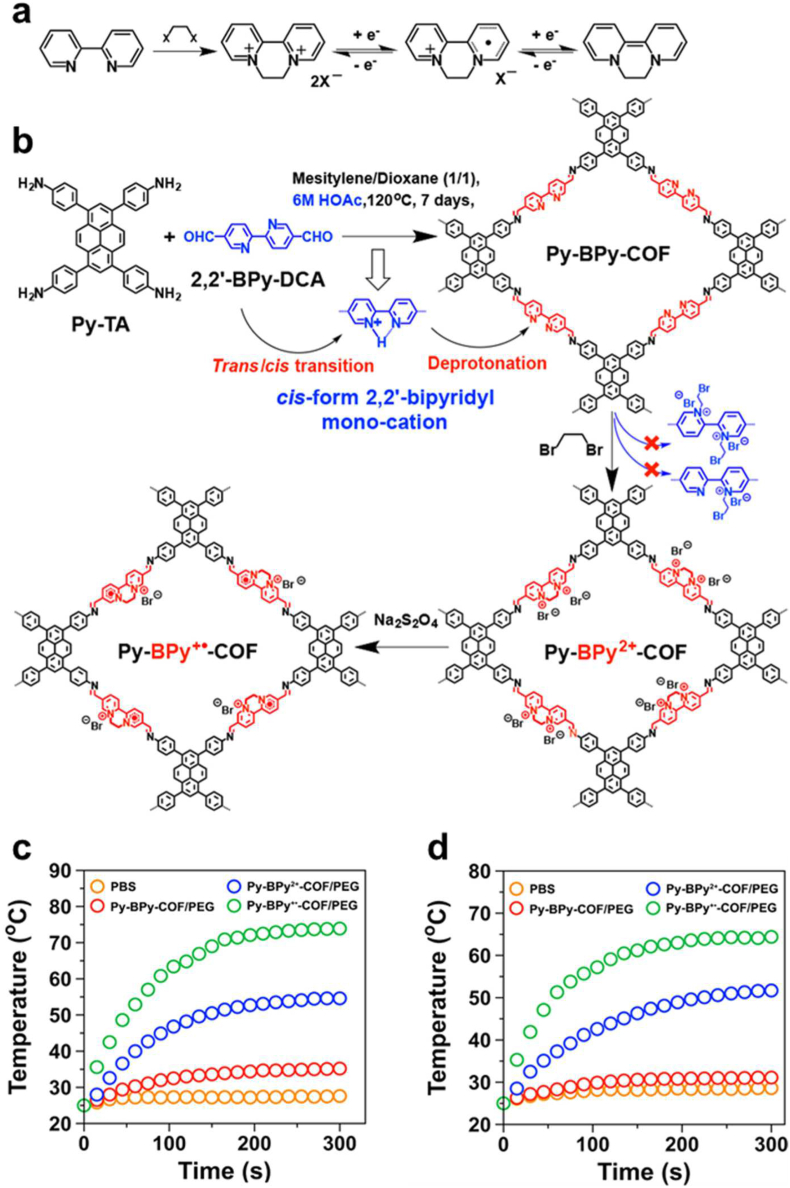
(a) Two reversible redox states of a diquat. (b) Transformation of Py-BPy-COF to cationic Py-BPy^2+^-COF and cationic radical Py-BPy^+•^-COF by two-step postmodification. During the process, the trans form of 2,2′-BPy-DCA is converted to the monocationic cis conformer in an acidic environment, enabling the formation of cyclic ethylated diquats, which could be further reduced with Na_2_S_2_O_4_. Elevated temperature change vs time for the dispersions of different PEG-modified COFs (100 μg/mL) and PBS as a control set upon exposure to 808 nm laser (c) and 1064 nm laser (d) for 5 min at a power of 1 W cm^−2^. Reproduced with permission from ref. 148. Copyright 2019, ACS publications.

In addition, Guo et al. presents a new strategy to convert 2,2′-bipyridine-based COF from neutral to positively charged and eventually to a cationic radical framework, allowing redox centers' superposition (Fig. 14) [148]. NIR absorption and photothermal conversion are achieved through the interchange transfer between π-coupling multilayers. Also, a structure-to-activity relationship has been established regarding the photothermal effect to generate an exceptionally high heat generation efficiency. The PEG-modified COFs and Fe_3_O_4_@COF(TpBD) showed appropriate biocompatibility in in-vivo applications.

**Fig. 15:**
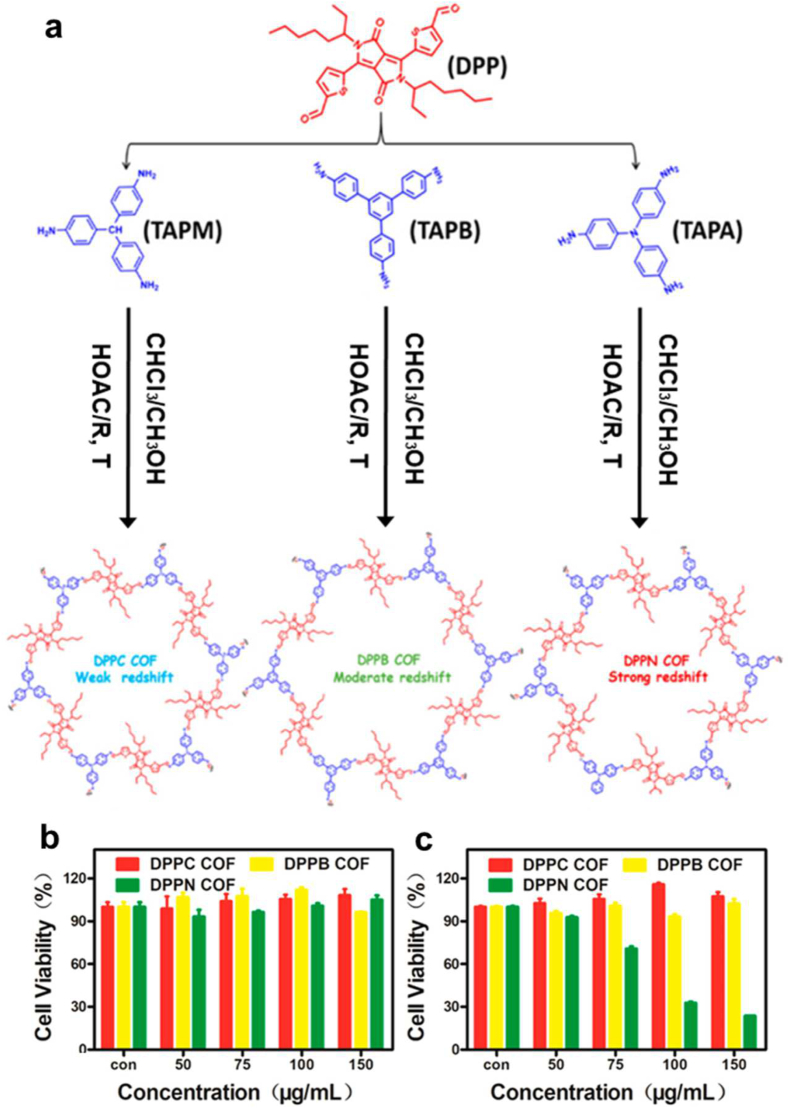
(a) Structures of amino and aldehyde monomers, and proposed units formed in the frameworks **(b)** Cytotoxicity of HeLa cells treated with DPPC, DPPB, and DPPN COF without laser irradiation **(c)** Cytotoxicity of HeLa cells treated with DPPC, DPPB, and DPPN COF with 808 nm laser (0.8 W cm^−2^) irradiation for 5 min. Reproduced with permission from ref. 149. Copyright 2021, ACS publications.

**Fig. 16:**
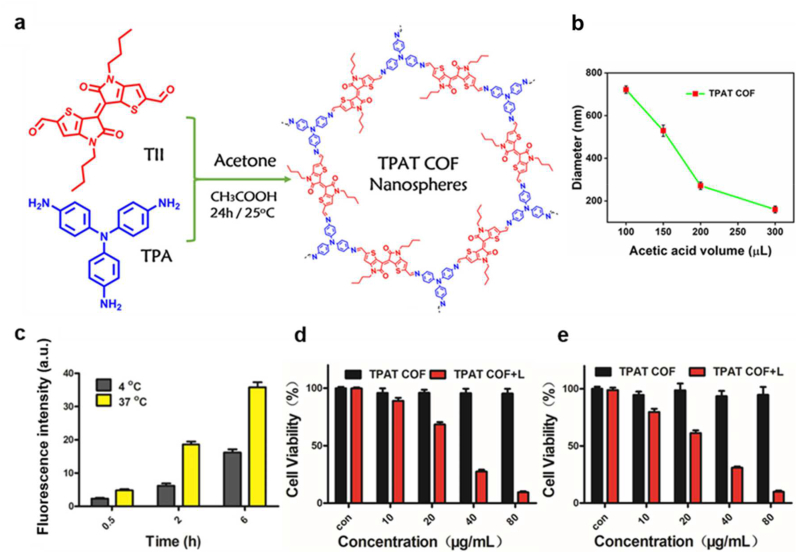
(a) Synthesis and characterization of COF. Monomers and the synthesis route of TPAT COF. **(b)** Diameter statistics with different acid dosages measured by DLS, and each sample is tested three times. **(c)** Fluorescence intensity statistics of HeLa cells incubated with TPAT COF/BDP at different time points and temperature (n = 3) *in vitro* cytotoxicity. Cell viability of TPAT COF against **(d)** HeLa and **(e)** HepG2 cells with 808 nm laser irradiation (0.75 W cm^−2^) (n = 3). Reproduced with permission from ref. 150. Copyright 2022, ACS publications.

In another example, Xia et al. presented donor acceptor-based COFs showing excellent fluorescence quantum yield due to extended conjugation, donors-acceptors combination, and separated HOMO and LUMO positions. They showed and compared the efficacy of each PDT by synthesizing three COFs using 5,5′-(2,5-bis(2-ethylhexyl)-3,6-dioxo-2,3,5,6-tetrahydropyrrolo- [3,4-c]-pyrrole-1,4-diyl) bis(thiophene-2-carbaldehyde) (DPP) as a core, DPPC DPPB DPPN combined with tris(4-aminophenyl)methane (TAPM), tris(4-aminophenyl)benzene (TAPB), and tris(4-aminophenyl)amine (TAPA) respectively. Among them, DPPN COF had the best PDT efficiency, possibly due to the electron-donating group (amine) in the middle of the molecule (Fig. 15) [149]. Furthermore, the same group introduced triphenylamine (TPA, electron donor) and thieno isoindigo (TII, electron acceptor) based COFs and named them TPAT COF (Fig. 16). [150]. They demonstrated the high-efficiency TPAT COF in PTT with laser irradiation (1.0 W cm^−2^, 808 nm, 5 min). Finally, DPPN COF and TPAT COF had good biocompatibility and PDT effect, as demonstrated by effective tumor suppression upon 808 nm laser irradiation.

### 5.6 COFs for CDT

Chemotherapy is still one of the most commonly prescribed cancer treatments [151,152]. However, multidrug resistance (MDR) has severely limited the therapeutic efficacy, with approximately 90% of patients encountering this issue during chemotherapy [[153], [154], [155]]. CDT causes tumor cells to die by catalytically converting internal hydrogen peroxide (H_2_O_2_) into the very lethal hydroxyl radical (·OH) through Fenton-like mechanisms [152,156]. CDT has been a successful anti-cancer approach in both *in vitro* and *in vivo* studies.

**Fig. 17:**
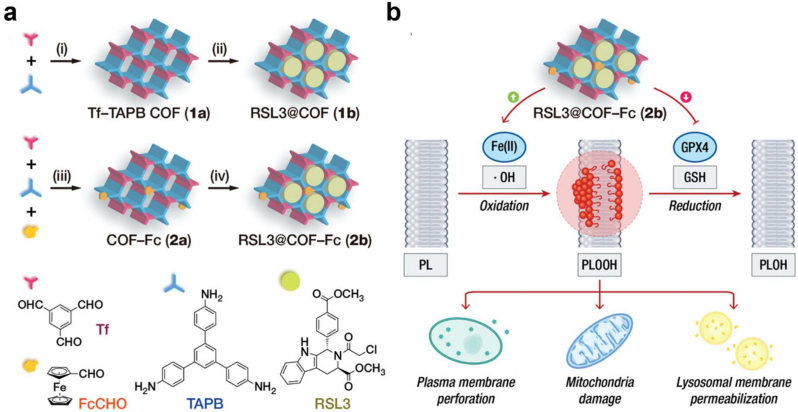
(a) Synthetic processes of COF-based nanomaterial for enhancing CDT via redox dyshomeostasis. **(b)** Enhanced cytotoxicity by inducing lipid peroxidation and blocking GPX4-mediated reduction of PLOOH. Reproduced with permission from ref. 158. Copyright 2021, Wiley-VCH.

Gao et al. prepared catalytically active Fe-porphyrin COF nanoparticles, **COF(Fe),** to overcome tumor MDR, which has a large capacity for drug loading [157]. Catalytic sites of **COF(Fe)** may effectively convert intracellular H_2_O_2_ that has been overexpressed into ·OH, forcing cancer cells to undergo oxidative damage and suppressing the production of the MDR-related protein P-gp. In addition, the DOX loaded COF(Fe), **DOX@COF(Fe)** had a substantial internalization impact in cells, allowing it to progressively release DOX in an acidic intracellular environment and demonstrating remarkable anti-cancer effects *in vitro* as well as *in vivo*. Moreover, Zhou et al. developed **RSL3@COF–Fc (2b)**, which comprises ferrocene (Fc) and glutathione peroxidase 4 (GPX4) inhibitors to accelerate CDT-induced cellular damage by ·OH (Fig. 17) [158]. They introduced a new redox dyshomeostasis treatment method to examine the COF-based nanomaterials' potential in enhancing CDT effectiveness. After tumor cells' endocytosis of **2b**, RSL3 was released to block GPX4, an essential stage in intracellular lipid repair, compromising intracellular redox equilibrium. At the same time, Fc-induced ·OH generation causes permanent ferroptotic cell death. **2b** eventually led cancer cells to lose their plasma membranes, lysosomes, and mitochondria, leading to ferroptosis while being less hazardous to normal cells.

### 5.7 COFs in combination therapy

**Fig. 18:**
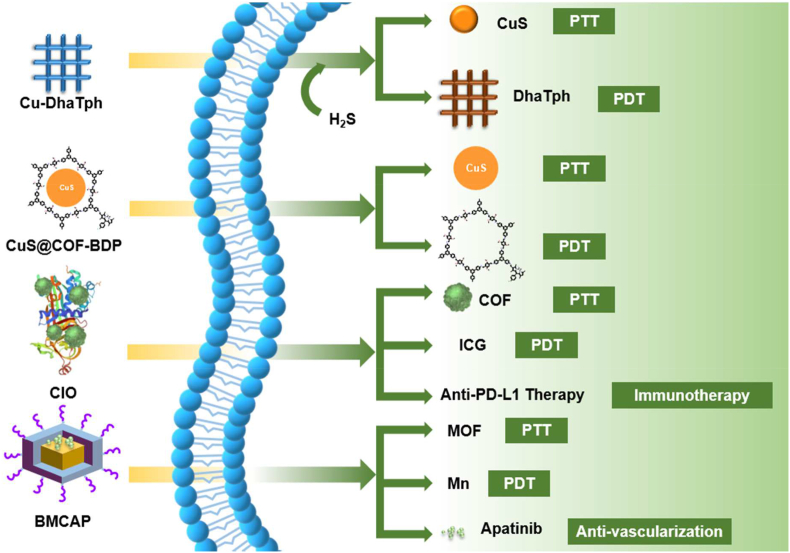
Schematic illustration of various types of combination therapy.

Among various therapeutic methods to cure cancer, PDT is highly efficient for solid tumor and local cancer treatment [159]. Although it has undergone significant advancement in cancer therapy, many limitations hold its potential; similarly, other cancer therapies also have limitations in contrast to expected results [160]. For example, PTT demands high selectivity unless all cells are malignant. Heat could damage normal cells, making complete tumor ablation impossible [161]. Similarly, other methods, such as PDT, immunotherapy, microwave-mediated therapy, etc., also have obstacles to be solved [[162], [163], [164], [165]]. Researchers have been dealing with the limitations and in 2019, Chen et al. devised COF possessing a dual-modal function [166]. They exploited COF as a PTT agent and a drug carrier to enhance dispersibility and water stability. The COF was used to increase the water solubility of drugs in the presence of PDT/PTT abilities in Dong's group [167]. Adopting combination therapy using multiple therapeutic strategies has been quite successful in the synergetic and effective elimination of cancer (Fig. 18).

**Fig. 19:**
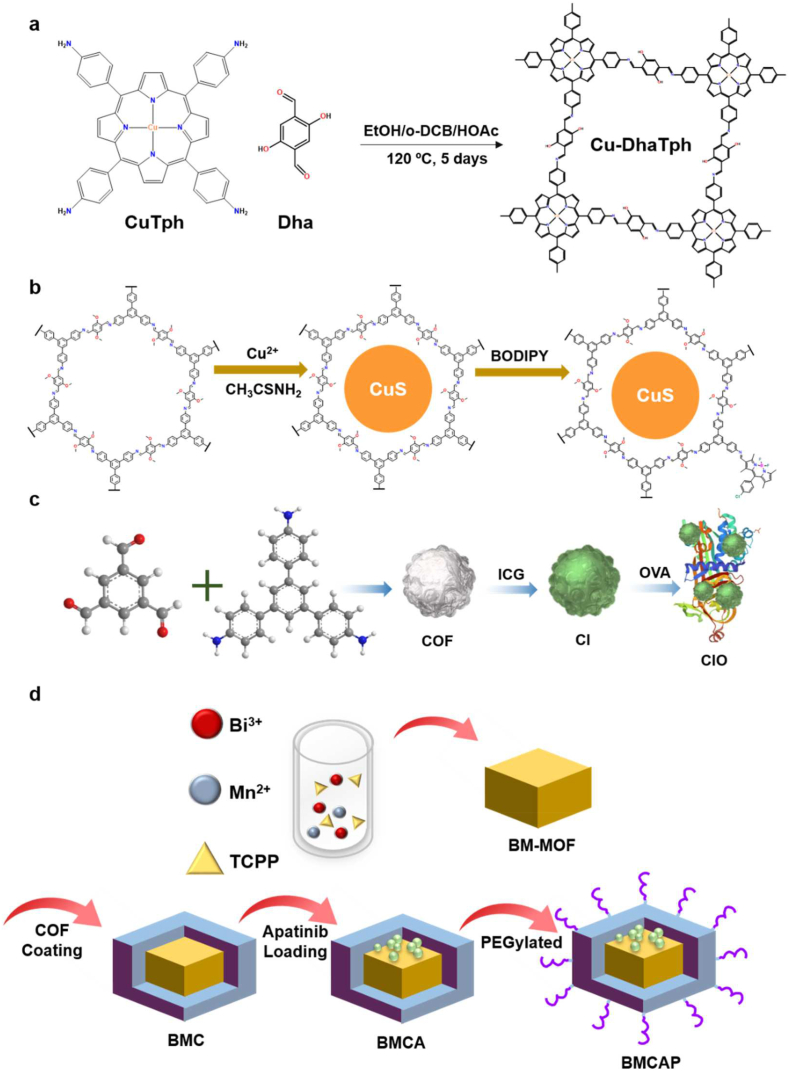
(a) Synthesis of endogenous H_2_S-activated nano Cu-DhaTph for effective *in situ* phototherapy. (b) Schematic illustration of the preparation of CuS@COF-BDP for combined PDT and PTT treatment. (c) Fabrication of CIO nanoparticle for phototherapy combined with checkpoint blockade immunotherapy. (d) Schematic diagram of synthesis of BMCAP nanocapsule for PDT and PTT induced by MW.

Several COFs have previously been applied in various cancer therapies with limited to outstanding efficacies and good enough biocompatibility in cellular microenvironments [[163], [164], [165], [166], [167], [168], [169], [170]]. Recently Dong et al. introduced Cu-DhaTph COF, a dual-functionalized anti-cancer agent (Fig. 19a) [148]. They combined Cu(Ⅱ), a highly selective responding species with H_2_S, with metal-free COF(DhaTph). The average size of Cu-DhaTph was 75 nm, and the H&E staining at the heart, liver, spleen, lung, and kidney demonstrated competence for biocompatibility. When it gets into the cell, the Cu(Ⅱ) ion reacts with H_2_S forming a photothermal conversion agent, CuS. The remaining COF(DhaTph) acts as a PDT agent simultaneously to produce ^1^O_2_ after the sulfidation reaction. To authenticate the sequential process, Cu-DhaTPh releases the photosensitizer of DhaTph. They demonstrated the combination of PDT and PTT performs synergetic cancer-killing in the microenvironment. Soon after this encouraging result, Dong's group developed another combination therapy using modified COF. In this article, the authors replaced the CuS strategy with the BODIPY derivatives (Fig. 19b) [162]. They modified the CuS@COF by connecting the BODIPY derivative with the –NH_2_ end groups of the COF to get CuS@COF-BDP. CuS@COF-BDP NPs showed good biocompatibility with particles size around 140 nm. The authors also checked the stable binding of BODIPY with CuS@COF-BDP under physiological conditions. CuS@COF-BDP efficiently worked as a dual-modal therapeutic agent to selectively inhibit MCF-7 cancer cells by its photothermal efficiency and efficient ^1^O_2_ generation. They also tested the cell viability of this COF material that remained intact after 24 h at the concentration of 200 μg/mL COF.

In 2020, Zhou et al. combined tumor-associated antigens (TAA) to stimulate antitumor immune response after dual photo therapy, PDT and PTT [164]. The COF prepared using TAPB and 1,3,5-benzenetricarbaldehyde (BTCA) was modified with FDA-approved photosensitizer ICG via surface absorption to get COF@ICG (Fig. 19c). COF@ICG was further coated with chicken ovalbumin (OVA) to get COF@ICG@OVA. The particle size of final nanomaterial COF@ICG@OVA was 100 nm and showed good biocompatibility up to 200 μg/mL concentration. As a combination therapy model, they used PDT from COF and PTT from ICG followed by conjugation of anti-PD-L1 checkpoint blockade. As a result, the complementary usage of multiple therapies dramatically decreases cancer metastasis and induces systemic immunity. Like the improved stability and biocompatibility of ICG at Pang's application, the anti-angiogenesis drug, Apatinib is carried out using COF by Li et al. [165]. They used MOF@COF nanocapsule (BMCAP) consisting of Bi^3+^, Mn^2+^, and TCPP, which could generate heat and ROS by microwave irradiation (Fig. 19d). They suggested a hydrated particle size of about 160 nm with good biocompatibility with cells at 200 μg/mL for 24 h. Hence, the nanocapsule interacted with the anti-angiogenic drug through strong π-π conjugation. The multi-functional attacks diminish the tumor size significantly, implying the efficiency of the combinational strategy. COF could mediate several therapies in one reagent, and complementary results lead to synergetic results. In combination therapy, systematically designed and modified COFs could act as PDT agents and drug carriers with a high degree of biocompatibility. Like MOFs, COFs can also be constructed with analogous permanent porosity and other advantageous thermo-optical properties for cancer therapies. Additionally, COFs offer better biocompatibility in combination therapy due to their metal-free fully conjugated network. Also, the fully conjugated network of COF can be modified within a minimal percentage of metals to enhance PDT, CDT and PTT efficiency [[165], [166], [167], [168], [169], [170]].

## 6 Conclusions and perspective

In summary, we reviewed the biocompatibility of recently reported COF nanomaterials applied in drug delivery, phototherapy, photo-immunotherapy, cancer imaging-guided therapy, chemotherapy and pyroptosis applications. We recapped the several intrinsic properties of COF NPs that make them advantageous for cancer theranostic applications in this category. Generally, hydrothermally synthesized COFs have poor biocompatibility in terms of NPs size, morphology, solubility, and dispersibility in biological media, resulting in weak cellular uptake. Recently developed pre- and post-synthetic modifications of COF have presented compatible morphologies and improved dispersibility in physiological media that considerably enhances cellular uptake. PEGylation or modifications with hydrophilic chains further improves the dispersibility. The surface coating of COFs on the other regular NPs has also shown good cell uptake and enhanced efficacy in PDT and drug delivery. Also, recently developed green and eco-friendly synthesis procedures have further reduced the chance of toxicity. The challenge of long-term dispersibility and crystallinity still needs to be addressed. Primarily, COFs or modified COFs have been found suitable to study cancer theranostic applications with some degree of toxicity. However, the long-term toxicity effect has yet to be explored in detail. We also discussed current challenges and future strategies to optimize COF-based nanomaterials to take them to clinical trials.

1. Several advanced synthetic protocols have now been developed to prepare COF NPs with suitable biocompatibility for various anti-cancer drug loading and targeted delivery by disintegration at a relatively lower pH of the cancer microenvironment. *In vivo* studies on mouse models have suggested that COF nanoparticles bypass biological barriers and accumulate at the tumor site effectively. COFs have shown tremendous potential as carriers of anti-cancer drugs, photothermal agents and photosensitizers with high loading efficiency and selective delivery to the tumor location.
2. To overcome the clinical limitation of molecular PSs, such as their poor localization at the tumor site, limited penetration depth, dark toxicity etc., several COF NPs have been engaged in phototherapy applications. The admirable biocompatibility of nCOFs has ensured their effective localization at the tumor site. Band gap engineering in donor-acceptor-based nCOFs has displayed adequate light absorption in the NIR region and improved PDT and PTT effects in in-vivo studies. With the high crystallinity and excellent photophysical properties, PDT efficiency of COFs is continuously enhancing. Recently developed nCOFs with an ability of two-photon absorption in the NIR region have presented enhanced efficacy at lower energy excitation with higher penetration depth. A few examples of nCOFs have also induced immunological memory in the mouse model of breast cancer that lasted as many as 110 days. These results suggest that nCOFs can attain good biocompatibility via proper modifications for high PDT efficacy with long-term immunological memory. Adequate ROS generation capacity of metal-modified nCOFs has also shown high efficacy in SDT to treat deeper tumors.
3. Conventional post-synthetic modifications of COFs derivatized them to work as cancer imaging agents or PDT agents. Pore volume optimization simply by changing the building blocks makes COFs fit for loading any desired anti-cancer drugs. These unique properties make them a suitable candidate for multi-functional combination therapy agents.
4. We have reviewed and discussed some reported facts regarding the biocompatibility of nCOFs. Initially discovered solvothermal synthesis of COFs mostly results in hydrophobic, larger nanoparticles that are hardly dispersible in aqueous media, hence were not suitable for biomedical applications. In past few years pre and post-synthetic methods have been developed to produce smaller (50–200 nm), hydrophilic nanoparticles and have been successfully applied in anti-cancer applications. The recent literature has shown the way to solve the fundamental difficulties in scaling up synthesis and designing COFs of compatible sizes, however the standard protocols to formulate biocompatible nCOFs are still in the developing stage. The intense in-vivo research started only recently that need to be further tested for sustainability. Although it seems a long way to go in developing safe nCOFs-based anti-cancer nanomedicines but recent results are promising for taking them to clinical trials.

COF NPs for desired functions and geometry are designed simply by choosing the building blocks of required dimensions and functional groups. Shortly, nCOFs are anticipated to come in the successful targeted delivery of multi-nano-sized drugs and biomolecules. Several COF-based nanomedicines are actively explored in in-vivo cancer diagnosis and treatment. Although the current state of COF NPs in theranostic applications is exciting and nCOFs have the potential to enhance further the drug loading and cancer cell targeting ability. Recently discovered photo-immunotherapy and pyroptosis applications of COF are promising; there is enormous potential for further in-depth research in this area. Pre- and post-synthetic modifications of nCOFs are continuously enhancing the biocompatibility and efficiencies in radiation-based therapies owing to the possibilities of COFs for simple transformation via stable covalent bonds. The future research focused on advancement of near IR active stable nCOFs to enhance efficiency, selectivity and penetration depth in radiation therapies will have an upper hand in overcoming existing limitations. The upcoming research should also be focused on developing biomolecules modified COF NPs with cell organelles targeting abilities for better cell uptake properties and enhanced therapeutic efficacy. It is high time to see the further in-depth *in vivo* and long-term toxicity studies to take COFs to clinical trials. The promising unique properties of COFs and the advantages of modified COFs will soon open new ways to improve cancer theranostics efficacy without significant toxicity. The collective advances in rational design will make them potential contenders for clinical trials.

## Ethics approval and consent to participate

The manuscript has no reporting involving human participants.

## Declaration of competing interest

The authors declare no conflict of interest.
